# Modulation of mTOR and CREB pathways following mGluR5 blockade contribute to improved Huntington’s pathology in *zQ*175 mice

**DOI:** 10.1186/s13041-019-0456-1

**Published:** 2019-04-08

**Authors:** Khaled S. Abd-Elrahman, Stephen S. G. Ferguson

**Affiliations:** 1 University of Ottawa Brain and Mind Institute, University of Ottawa, 451 Smyth Road, Ottawa, Ontario K1H 8M5 Canada; 2 Department of Cellular and Molecular Medicine, University of Ottawa, 451 Smyth Road, Ottawa, Ontario K1H 8M5 Canada; 3 Department of Pharmacology and Toxicology, Faculty of Pharmacy, Alexandria University, Alexandria, 21521 Egypt

**Keywords:** mGluR5, Huntington’s disease, *zQ*175, mHTT, CTEP, ULK1, mTOR, BDNF, autophagy

## Abstract

Huntington’s disease (HD) is a neurodegenerative disorder caused by a genetic abnormality in the huntingtin gene that leads to a polyglutamine repeat expansion of the huntingtin protein. The cleaved polyglutamine expansion of mutant huntingtin (mHTT) protein can form aggregates strongly correlated with HD progression. We have previously shown that the inhibition of mGluR5 using CTEP, a selective negative allosteric mGluR5 modulator, can delay disease progression and reduce in mHTT aggregates in the *zQ*175 mouse model of HD. This was paralleled by enhanced catalytic activity of Unc-51-like kinase 1 (ULK1), a kinase modulated by mammalian target of rapamycin (mTOR) and key regulator of autophagy initiation. In the present study, we show that CTEP can correct aberrant phosphoinositide 3-kinase (PI3K)/Akt/mTOR signaling detected in *zQ*175 mice that may underlie the enhanced ULK1 activity and activation of autophagy. We also show that CTEP can facilitate cAMP response element-binding protein (CREB)-mediated expression of brain-derived neurotrophic factor (BDNF) to foster neuronal survival and reduce apoptosis. Taken together, our findings provide the molecular evidence for how targeting mGluR5 using a well-tolerated selective NAM can mitigate two critical mechanisms of neurodegeneration, autophagy and apoptosis.

## Introduction

Huntington’s disease (HD) is an adult-onset, inherited autosomal dominant neurodegenerative disorder caused by a polyglutamine (CAG) repeat expansion in exon 1 that encodes the amino-terminal of the huntingtin protein [1, 2]. It is characterized by progressive motor, cognitive psychiatric deficits and early mortality [3, 4]. Cleavage of the polyglutamine expanded amino terminus of huntingtin protein leads to the formation of intranuclear and cytoplasmic aggregates that are strongly correlated with HD onset and severity of symptoms [2, 5, 6]. To date, disease-modifying treatments for HD are lacking, which supports the necessity of identifying novel disease-altering mechanisms that can be targeted to slow the progression of HD.

Metabotropic glutamate receptor 5 (mGluR5) is a member of the Gα_q/11_ protein-coupled receptor family and is highly expressed in striatum and cortex, regions of the brain that are most affected in HD [7, 8]. Moreover, the genetic deletion of mGluR5 reduced mutant huntingtin (mHTT) aggregates size and improved disease pathology in a *Q111* knock-in mouse model of HD [9]. Thus, it is evident that targeting mGluR5 signaling can alter the accumulation mHTT aggregates and ameliorate HD pathology. Recently, we showed that the pharmacological blockade of mGluR5 using the selective negative allosteric modulator (NAM), CTEP, results in delayed disease progression and the reduction in mHTT aggregates found in the brains of a *zQ*175 knock-in mouse model of HD [10]. CTEP (2-chloro-4-[2[2,5-dimethyl-1-[4-(trifluoromethoxy) phenyl] imidazol-4-yl] ethynyl] pyridine) was chosen for this study because of its oral bioavailability, ability to cross the blood brain barrier, and extended half-life of 18 h [11]. Its analogue Basimglurant was proven to be well- tolerated in phase II trials for major depressive disorder [11, 12].

The favorable outcomes of mGluR5 blockade in the *zQ*175 model of HD and both the APPswe/PS1ΔE9 and 3xTg-AD models of Alzheimer’s disease were associated with increased autophagy via alterations in Zinc finger and BTB domain-containing protein 16 (ZBTB16)- and Unc-51-like kinase 1 (ULK1)-dependent mechanisms [10, 13]. Specifically, we showed that mGluR5 inhibition in *zQ*175 reduced ubiquitin-mediated degradation of the autophagy adaptor ATG14 via GSK3β-dependent inhibition of ZBTB16-Cullin3-Roc1 E3-ubiquitin ligase complex. Interestingly, CTEP also reduced the inhibitory phosphorylation of ULK1 at S757 that was paralleled by enhanced phosphorylation of the autophagy factor ATG13, required for autophagosome formation [10, 14]. Although the activation of ULK1 is key for autophagy initiation, the molecular cascade that is required to transduce the mGluR5 signaling to ULK1 remains poorly-defined [15]. A reduction in neuronal apoptosis and rescue of neurons when stained for neuronal nuclei (NeuN) in CTEP-treated *zQ*175 mice was also observed [10]. Since mHTT is known to alter transcriptional regulation and apoptosis [16–18], it remains unclear whether the autophagic clearance of mHTT following chronic mGuR5 inhibition can reduce the loss of striatal neurons and nurture the neurotrophic capacity in HD brains.

Here, we show that pharmacological antagonism of mGluR5 abolishes the enhanced phosphoinositide 3-kinase (PI3K)/Akt/mammalian target of rapamycin (mTOR) signaling observed in *zQ*175 mice. Specifically, CTEP reverses the elevated phosphorylation of phosphoinositide-dependent kinase-1 (PDK1), Akt and mTOR in *zQ*175 mice that may underlie the previously-reported reduction in inhibitory phosphorylation of ULK1 at S757 resulting in autophagy activation. The inhibition of mGluR5 in *zQ*175 mice is also associated with enhanced cAMP response element-binding protein (CREB) activity as well as cFos expression and Brain-derived neurotrophic factor (BDNF) synthesis. These findings provide a mechanistic link between mGluR5 signaling and ULK1 activity via PI3K/Akt/mTOR. It also indicates that the clearance of mHTT may influence CREB/cFos-mediated expression of BDNF to reduce apoptotic neuronal loss.

## Results

### Chronic mGluR5 antagonism normalizes mTOR activity in *zQ*175 mice

**Fig. 1:**
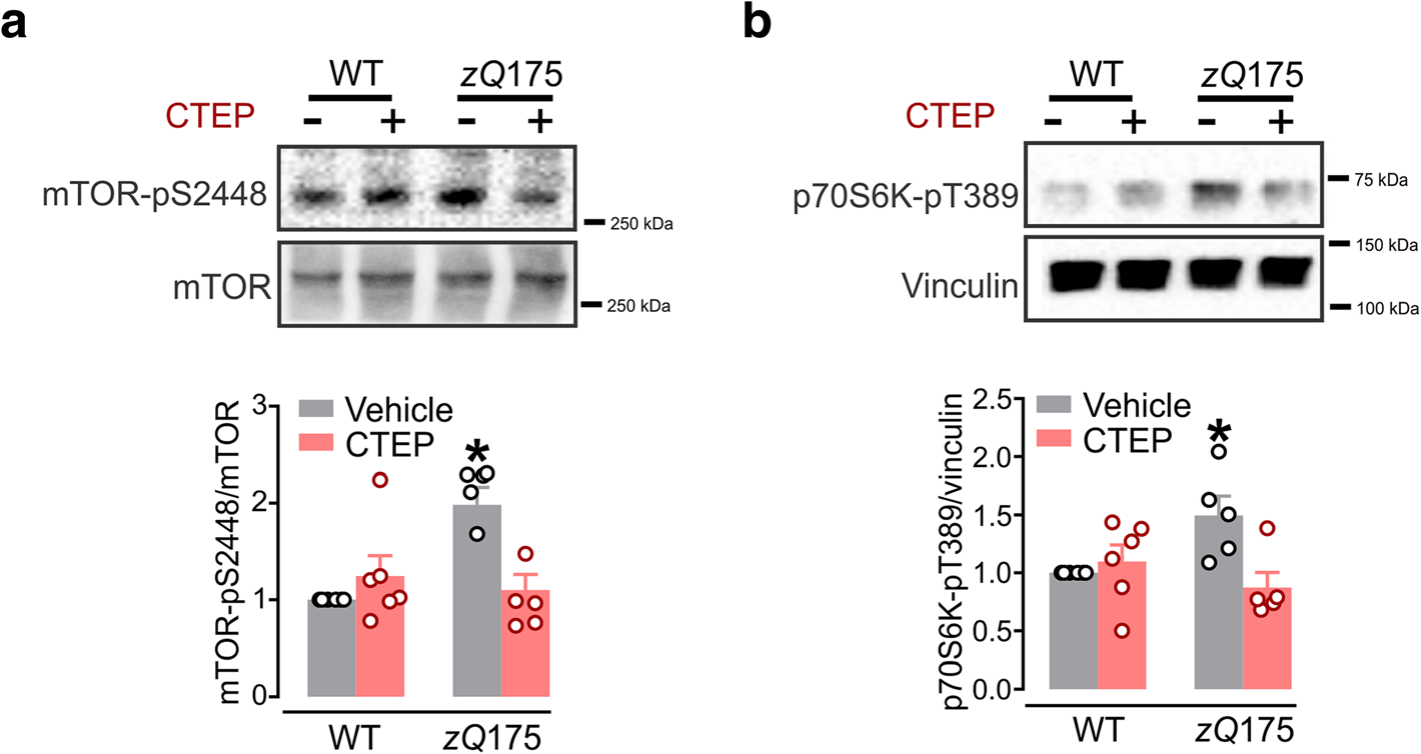
CTEP normalizes enhanced mTOR activity in *zQ*175 mice. **a** Representative western blots and mean ± SEM of mTOR-pS2448 and (**b**) p70S6K-pT389 in brain lysates from homozygous *zQ*175 and wildtype (WT) mice after chronic treatment with either vehicle or CTEP (2 mg/kg). Values are expressed as a fraction of the vehicle-treated WT. mTOR-pS2448 was normalized to total mTOR expression and p70S6K-pT389 was normalized to vinculin expression (*n* = 5–6 for each group). * *P* < 0.05 significantly different from vehicle-treated WT mice

A critical step in autophagy is the formation of the autophagosome and this step is primarily regulated by ULK1 [15]. Phosphorylation of ULK1 at S757 site by the mTOR complex results in suppression of its catalytic activity and inhibition of autophagy [15, 19]. We have previously reported that chronic inhibition of mGluR5 reduces the inhibitory phosphorylation of ULK1 at S757 site to induce autophagy [10]. Here, we tested whether mTOR activity was elevated in homozygous *zQ*175 mice that might explain reduced ULK1 activity and autophagy inhibition and whether mTOR activity can be modulated by CTEP. The phosphorylation of mTOR at S2448 has been demonstrated to represent the activation state of the PI3K pathway, in addition to serving as a biomarker for the activation status of mTOR [19–22]. Thus, we first examined changes in mTOR-pS2448 phosphorylation status in homozygous zQ175 huntingtin knock-in wildtype (WT) mice following a 12-week treatment with either vehicle or CTEP (2 mg/kg) at 12-months of old age. Brain lysates derived from vehicle-treated homozygous *zQ*175 mice showed a significant increase in mTOR-pS2448 phosphorylation compared with WT mice (Fig. 1a). Interestingly, the increase in mTOR-pS2448 phosphorylation was reversed in CTEP-treated homozygous *zQ*175 mice to values indistinguishable from WT. To further confirm that the changes in mTOR-S2448 phosphorylation reflected changes in mTOR signaling, we measured phosphorylation of the mTOR downstream ribosomal protein S6 kinase (p70S6K1). mTOR has been shown to regulate the protein translational machinery at synapses by modulating p70S6K1 activity through the direct phosphorylation at T389 and this phosphorylation has been considered to be a hallmark of mTOR activity [20, 23, 24]. Similar to mTOR-pS2448 phosphorylation, we detected higher levels of p70S6K1-pT389 phosphorylation in vehicle-treated homozygous *zQ*175 mice when compared to WT mice and we found that CTEP normalized the level of p70S6K1 phosphorylation in homozygous *zQ*175 when compared to WT mice (Fig. 1b). Taken together, these results indicated that chronic antagonism of mGluR5 with a selective NAM can correct the aberrant activation of mTOR pathway to trigger ULK1 activation and initiate autophagy.

### PI3K/Akt pathway plays a role in altered mTOR signaling of *zQ*175 mice

**Fig. 2:**
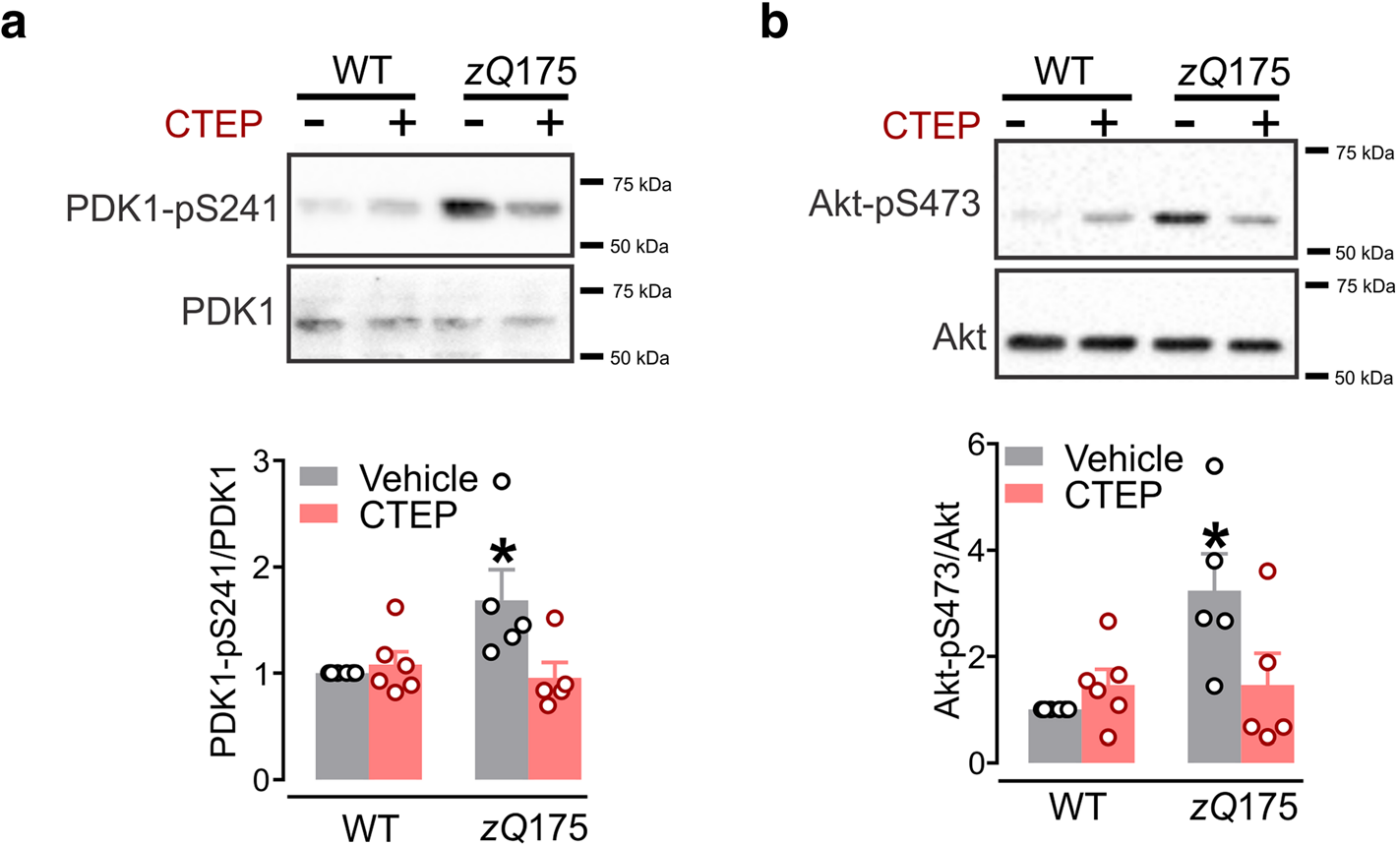
CTEP reverses abnormal PI3K/Akt signaling in *zQ*175 mice. **a** Representative western blots and mean ± SEM of phosphoinositide-dependent kinase-1 (PDK) PDK1-pS241 and (**b**) Akt-pS473 in brain lysates from homozygous *zQ*175 and wildtype (WT) mice after chronic treatment with either vehicle or CTEP (2 mg/kg). Values are expressed as a fraction of the vehicle-treated WT. PDK1-pS241 was normalized to total PDK1 expression and Akt-pS473 was normalized to total Akt expression (*n* = 5–6 for each group). * *P* < 0.05 significantly different from vehicle-treated WT mice

mTOR has been considered to be a key regulator of growth and autophagy and is activated downstream of PI3K. Specifically, phosphorylation phosphatidylinositol-3,4,5-trisphosphate by PI3K recruits and activates both PDK1 and Akt via direct phosphorylation that has been shown to mediate the activation of mTOR signaling [22, 25, 26]. Interestingly, activation of group I mGluRs has also been found to activate PI3K/Akt/mTOR signaling pathway in mouse hippocampus [27, 28]. Therefore, we assessed whether mGluR5 regulate mTOR and ULK1 signaling via the PI3K/PDK1/Akt signaling cascade. We detected a significant increase in the levels of PDK1-pS241 and Akt-pS473 in vehicle-treated homozygous *zQ*175 mice when compared to WT mice (Fig. 2a and b). Chronic inhibition of mGluR5 using CTEP abrogated the extent of PDK1-pS241 and Akt-pS473 activation in homozygous *zQ*175 mice such that the phosphorylation of these enzymes was indistinguishable from that of WT mice (Fig. 2a and b). Taken together, these results indicate that alterations in mGluR5-medaited PI3K/PDK1/Akt signaling can influence mTOR activity to modulate autophagy in *zQ*175 mice.

### Activation of CREB/cFos pathway following mGluR5 inhibition in *zQ*175 mice

**Fig. 3:**
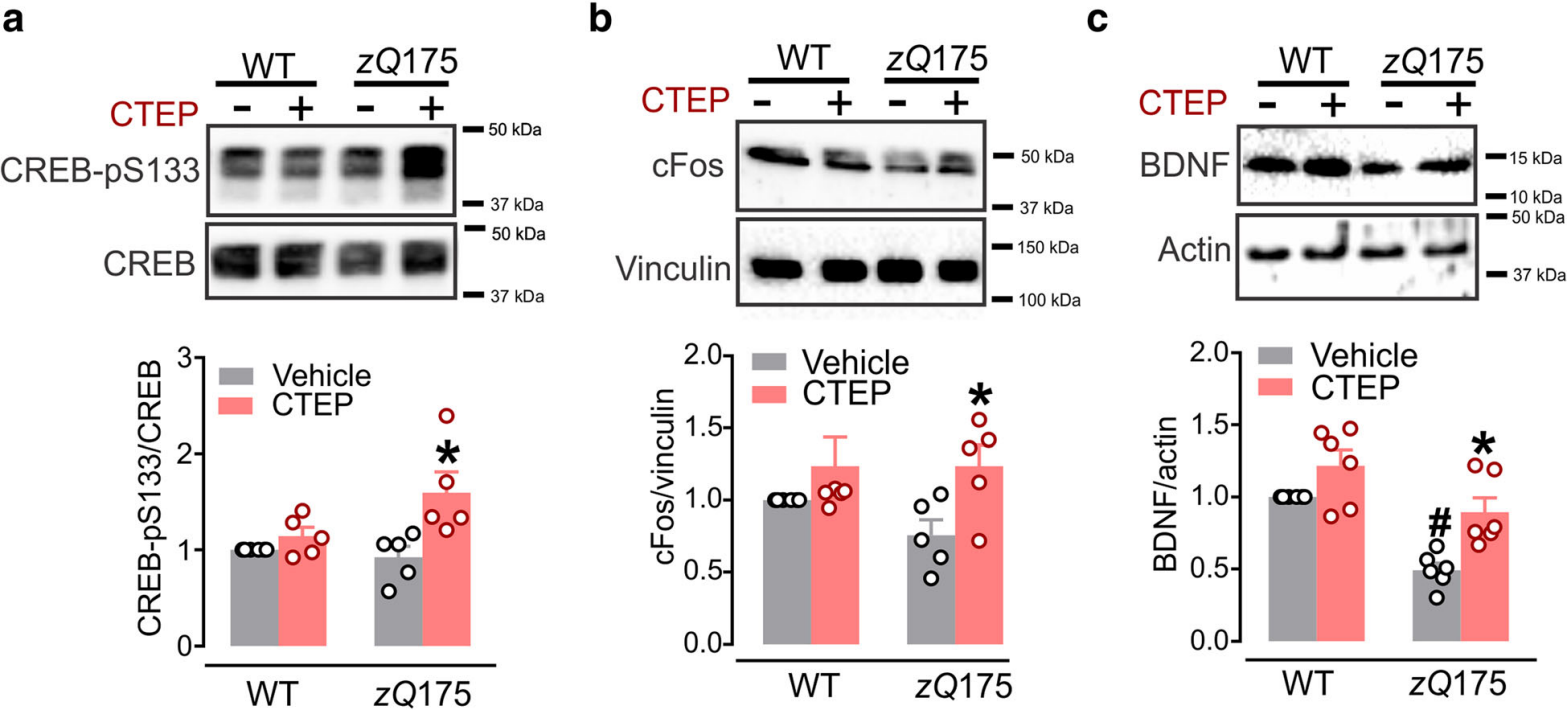
CTEP facilitates CREB-mediated expression of BDNF in *zQ*175 mice. **a** Representative western blots and mean ± SEM of CREB-pS133 (**b**) cFos and (**c**) BDNF in brain lysates from homozygous *zQ*175 and wildtype (WT) mice after chronic treatment with either vehicle or CTEP (2 mg/kg). Values are expressed as a fraction of the vehicle-treated WT. CREB-pS133 was normalized to total CREB, cFos was normalized to vinculin expression and BDNF was normalized to actin expression (n = 5–6 for each group). Representatives for each panel were obtained from the same blot. P < 0.05 * significantly different from vehicle-treated *zQ*175 mice and ^#^ P < 0.05 significantly different from vehicle-treated WT mice

CREB is a constitutive transcription factor that modulates the expression of various inducible transcription factors including c-Fos [29, 30]. BDNF is a neurotrophic factor that can support neuronal survival and differentiation and its expression is tightly regulated by cFos [31, 32]. Notably, mutant Htt aggregates sequesters CREB-binding protein (CBP), an important activator of CREB, to decrease the expression of CREB target genes [33, 34]. We previously reported that chronic mGluR5 inhibition in *zQ*175 partially reversed neuronal apoptosis and increased the number of surviving NeuN-positive striatal neurons in zQ175 mice [10]. Therefore, we tested whether CTEP-mediated activation of autophagy and reduction in mHTT aggregates was associated with enhanced CREB-dependent transcription of BDNF that could be associated with the rescue of neuronal loss in *zQ*175 mice in a CREB- and cFos-dependent manner. We found that CTEP treatment of zQ175 mice resulted in a significant increase in CREB-pS133 and cFos protein expression when compared to vehicle treated WT and homozygous zQ175 mice as well as CTEP-treated WT mice (Fig. 3a and b). We observed that BDNF expression was significantly reduced in vehicle-treated zQ175 mice when compared to either vehicle or CTEP treated WT mice and that CTEP treatment increased BDNF expression levels in homozygous zQ175 mice to WT levels (Fig. 3c). Thus, CTEP treatment was able to enhance BDNF synthesis in *zQ*175 mice and suggests that mGuR5 antagonism-mediated increase in mHTT autophagy might facilitate CREB/cFos/BDNF signaling to promote neuronal survival in HD mice.

## Discussion

We have demonstrated in a series of studies that the genetic and pharmacological silencing of mGluR5 represents a successful approach to slow HD progression and reverse HD pathology [9, 10]. Specifically, the mGluR5 NAM CTEP displays an intrinsic capacity of slowing disease pathology via autophagic clearance of mHTT aggregates and promoting survival of striatal neurons without the need to introduce potentially antigenic gene silencing agents [10, 35, 36]. Given the key role of mTOR in regulating autophagy, our findings from this study demonstrate an obligatory role of PI3K/Akt/mTOR pathway in modulating ULK1-dependent autophagy in *zQ*175 mice [37, 38]. Specifically, we show that PDK1/Akt/mTOR signaling is enhanced in *zQ*175 mice and this likely contributes to inhibition of ULK1 activity resulting in the reduced autophagic clearance of mHTT that we previously reported in zQ175 mice [10]. Chronic inhibition with CTEP rectified this altered PDK1/Akt/mTOR signaling and can now be associated with ULK1 activation and autophagy initiation. Moreover, we provide evidence that the reduction in the mHTT load following mGluR5 blockade is associated with an enhanced CREB/cFos-mediated expression of BDNF. This increase in BDNF expression is likely to contribute to the reduction in apoptotic loss of striatal neurons in *zQ*175 mice [10].

Autophagy plays a key role of in neuronal health by clearing cellular cargos and protein aggregates and defects in autophagy have been increasingly implicated in proteinopathies such as HD, Alzheimer’s and Parkinson’s disease [39–42]. This study extends our previous work using the mGluR5 NAM by identifying novel mGluR5-regulated signaling cascades that are required for ULK1 activation and autophagy initiation in *zQ*175 HD mice. We have previously reported that pharmacological inhibition of mGluR5 improved motor and cognitive deficits in the *zQ*175 mouse model of HD due activation of both ZBTB16- and ULK1-dependent mechanisms of autophagy [10]. The activation of the catalytic activity of ULK1 is due to a reduction in the inhibitory phosphorylation at S757 [15]. Interestingly mTOR, a key regulator of autophagy, is known to phosphorylate ULK1 at S757 [37, 43, 44]. Here we provide direct experimental evidence that mGluR5 inhibition modulates PI3K/Akt/mTOR signaling resulting in ULK1 activation and the initiation of autophagy. Canonical mTOR signaling is initiated following receptor-dependent activation of PI3K to phosphorylate PDK1 at S241 [26]. Active PDK1 directly activates Akt via phosphorylation that leads to the phosphorylation of mTOR at S2448 site [20, 26]. Thus, mTOR-pS2448 is considered a reliable indicator of the activation state of the PI3K pathway and mTOR complex [19–22]. Here, we show that CTEP can normalize the levels of PDK1-pS241, Akt-pS473 and mTOR-pS2448 in *zQ*175 mice. We also detected a reduction of phosphorylation of P70S6K1 at pT389, a kinase responsible for many of the consequences of mTOR downstream signaling and is considered a hallmark of mTOR activity [20, 23, 24]. It is worth noting that the activation of the PI3K/Akt/mTOR signaling cascade has been previously reported following agonist-dependent stimulation of mGluR5 and was required for mGluR5-dependent long term depression in mouse hippocampus [28]. Also, mHTT protein can bind and regulate different aspects of mGluR5 signaling [45, 46]. Thus, it is possible that in advanced HD stages mHTT enhances mGluR5 signaling via PI3K/Akt/mTOR pathway leading to autophagy inhibition and accumulation of mHTT aggregates that exacerbates HD pathology.

mGluR5 initiates a variety of signaling pathways via the canonical Gαq-coupled mechanism and concomitantly regulates gene expression at both the translational and transcriptional level to support neuronal survival, differentiation and synaptic plasticity [7, 47]. Our focus in this report is CREB, since its activity was found to be modulated by both mGluR5 and huntingtin protein [34, 47]. Upon activation by phosphorylation, p-CREB binds to the cAMP response element (CRE) site within the gene and triggers target gene transcription including cFos [29, 30, 48–50]. cFos has been found to regulate BDNF expression in vivo that promotes the survival of and differentiation of neurons [51]. Moreover, BDNF itself can induce cFos transcription in a feedforward cascade [52–55]. Interestingly, mHTT aggregates can sequester CBP and suppress CREB-mediated genes expression [33, 34]. In fact, suppression of CREB targeted genes is associated with early memory impairment memory in (Q7/Q111) HD mouse model [56]. Here, we show that the previously-reported attenuation in apoptosis and rescue of NeuN-positive striatal neurons in CTEP-treated *zQ*175 mice [10] is accompanied by enhanced CREB phosphorylation and, expression of cFos and BDNF. It is worth noting that we did not detect a significant change in CREB phosphorylation of cFos expression in CTEP-treated control mice indicating a pivotal role of mHTT in regulating CREB/cFos pathway in HD mice. Thus, it is likely that the autophagic clearance of mHTT facilitates CREB-dependent gene expression and amplify BDNF synthesis that can support neuronal survival and reduce apoptosis. Further experiments are required to detect whether the origin of synthesized BDNF is neuronal or glial and to confirm that the autophagic clearance of mHTT is key in regulating BDNF expression by pharmacologically blocking autophagy and measuring BDNF levels in our HD mice.

**Fig. 4:**
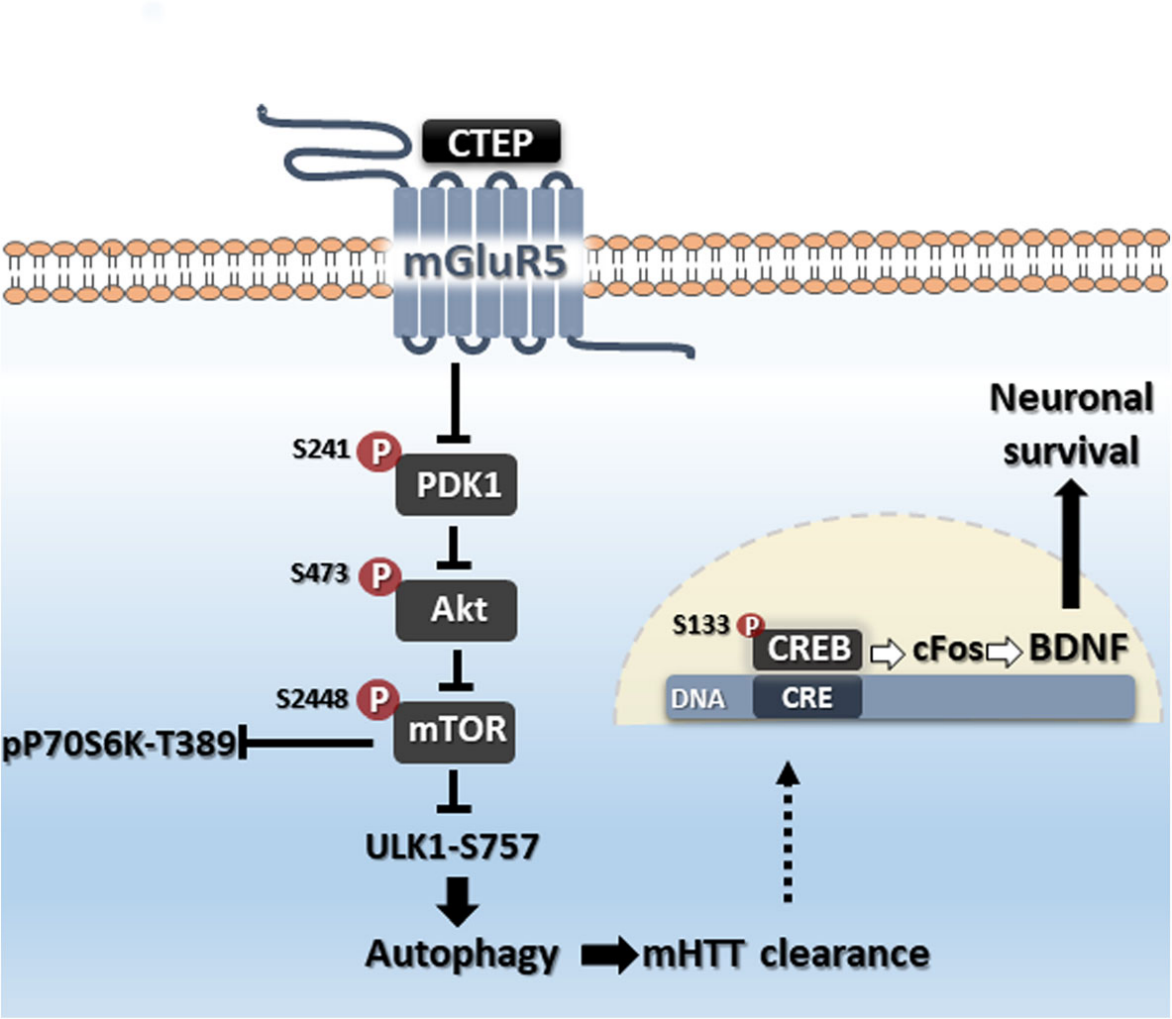
Schematic representation for how mGluR5 antagonism modulates mTOR and CREB signaling in *zQ*175 mice. The pharmacological inhibition of mGluR5 with CTEP in *zQ*175 mice abolishes the enhanced signaling of mammalian target of rapamycin (mTOR) by reducing the phosphorylation of phosphoinositide-dependent kinase-1 (PDK1), Akt and mTOR. Reduced mTOR signaling was confirmed by a reduction in the phosphorylation of downstream p70S6K and was associated with decreased inhibitory phosphorylation of ULK1 at S757 leading to activation of autophagy. Activation of autophagy and reduction in mHTT load can facilitate the binding of phosphorylated cAMP response element-binding protein (CREB) activity to cAMP response element (CRE) in the nucleus. Activation of CREB-mediated gene expression of cFos and brain-derived neurotrophic factor (BDNF) synthesis can contribute to neuronal survival and reduced apoptosis in zQ175 mice

As summarized in Fig. 4, we show that mGluR5 antagonism represents an effective approach to potentially halt HD progression by reversing mTOR-mediated inhibition of autophagy to reduce mHTT aggregates, and facilitate CREB-mediated expression of BDNF. Our data support the hypothesis that the mGluR5-dependent activation of mTOR pathway in advanced stages of HD is not favorable due to its inhibitory influence on ULK1 and autophagy leading to toxic accumulation of mHTT. We also provide evidence that the reduction of mHTT burden enhances CREB-mediated gene expression to support neuronal survival. We suggest that pharmacologically targeting mGluR5 via a well-tolerated selective NAM will be effective in slowing two mechanisms of neurodegeneration in HD, accumulation of neurotoxic aggregates and apoptotic neuronal loss. This report provides a better understanding of the pathophysiological signals in neurodegeneration and mechanism(s) that can be targeted by mGluR5 NAM and further supports their repurposing for treating neurodegenerative diseases.

## Materials and methods

### Reagents

CTEP was purchased from Axon Medchem. Horseradish peroxidase (HRP)-conjugated anti-rabbit IgG secondary antibody was from Bio-Rad (1662408EDU). Rabbit anti-actin (CL2810AP) was from Cedarlane (Burlington, Ontario). Mouse anti-BDNF (205067), rabbit anti-cFos (190289), anti-vinculin (129002) and anti-CREB (32515) antibodies were from Abcam (Cambridge, Massachusetts). Rabbit anti-phospho CREB-S133(06–519) and mouse anti-phospho P70 S6K-T389 (07–018-I) antibodies were from Sigma-Aldrich (St. Louis, Missouri). Rabbit Anti-phospho PDK1-S241(3438), anti-phospho Akt-S437(4060), anti-phospho mTOR-S2448 (109268), anti-PDK1 (3062), anti-mTOR (2972) and mouse anti-Akt (9272) from Cell Signaling Technology (Danvers, Massachusetts). Reagents used for western blotting were purchased from (Bio-Rad Laboratories, Hercules, California) and all other biochemical reagents were from Sigma-Aldrich (St. Louis, Missouri).

### Animals

All animal experimental protocols were approved by the University of Ottawa Institutional Animal Care Committee and were in accordance with the Canadian Council of Animal Care guidelines. Animals were individually housed under a constant 12 h light/dark cycle and given food and water ad libitum. Heterozygous *zQ175* HD mice were obtained courtesy of CHDI from Jackson laboratories, stock # 370476, and bred to establish littermate controlled male wild-type (WT), and homozygous *zQ*175 (*zQ*175*)* knock-in mice. *zQ*175 knock-in mice carry ~ 188 CAG repeat expansion. Groups of 12 male wild-type and *zQ*175 mice were aged to 12 months of age and 5–6 mice from each group were treated every 48 h with either vehicle (DMSO in chocolate pudding) or CTEP (2 mg/kg, dissolved in 10% DMSO mixed with chocolate pudding) for 12 weeks. This drug dose was calculated weekly based on weight and was shown to reverse motor and cognitive impairments in Huntington’s and Alzheimer’s mice [10, 57]. At the end of the 12-week treatment, mice were sacrificed by exsanguination and brains were collected and randomized for biochemical determinations.

### Immunoblotting

Brain hemispheres was lysed in 1.5 ml ice-cold lysis buffer (50 mM Tris, pH 8.0, 150 mM NaCl, and 1% Triton X-100) containing protease inhibitors (1 mM AEBSF, 10 μg/ml leupeptin, and 2.5 μg/ml aprotinin) and phosphatase inhibitors (10 mM NaF and 500 μM Na_3_VO_4_) and centrifuged at 15000 rpm at 4 °C for 15 min. The supernatant was collected and total protein levels were quantified using Bradford Protein Assay (Bio-Rad). Homogenates were diluted in a mix of lysis buffer and β-mercaptoethanol containing 3x loading buffer and boiled for 10 min at 95 °C. Aliquots containing 35 μg total proteins were resolved by electrophoresis on either 7.5% or 12% SDS-PAGE and transferred onto nitrocellulose membranes. Blots were blocked in Tris-buffered saline containing 0.05% of Tween 20 (TBST) and 5% non-fat dry milk for 2 h at room temperature and then incubated overnight at 4 °C with primary antibodies diluted 1:1000 in TBST containing 1% non-fat dry milk. Immunodetection was performed by incubating with secondary antibodies (anti-rabbit/mouse) diluted 1:5000 in TBST containing 1% of non-fat dry milk for 1 h. Membranes were washed in TBST and then bands were detected and quantified using BioRad chemiluminescence system.

### Statistical analysis

Means ± SEM are shown for each of independent experiments are shown in the various figure legends. GraphPad Prism software was used to analyze data for statistical significance. Statistical significance was determined by a series of 2 (strain) × 2 (drug treatment) ANOVAs followed by Fisher’s LSD comparisons for the significant main effects or interactions.

## Abbreviations

APPswe: APPswe/PS1ΔE9
ATG14: Autophagy-related protein 14
BDNF: Brain-derived neurotrophic factor
CBP: CREB-binding protein
CRE: cAMP response element
CREB: cAMP response element-binding protein
CTEP: 2-chloro-4-[2[2,5-dimethyl-1-[4-(trifluoromethoxy) phenyl] imidazol-4-yl] ethynyl] pyridine
HD: Huntington’s disease;
mGluR5: metabotropic glutamate receptor 5;
mHTT: Mutant Huntingtin
mTOR: mammalian target of rapamycin;
NAM: Negative allosteric modulator;
NeuN: Neuronal Nuclei
PDK1: Phosphoinositide-Dependent Kinase-1
PI3K: Phosphoinositide 3-Kinase
ULK: Unc-51-Like Kinase;
ZBTB16: Zinc finger and BTB domain-containing protein 16
